# К юбилею главного редактора журнала «Проблемы эндокринологии» академика РАН Ивана Ивановича Дедова

**Published:** 2026-03-07

**Authors:** статья Редакционная

## Abstract

12 февраля 2026 г. отмечает юбилей Полный кавалер ордена «За заслуги перед Отечеством», академик РАН, выдающийся ученый с мировым именем Иван Иванович Дедов — ведущий клиницист-эндокринолог, педагог, опытный организатор здравоохранения России.

12 февраля 2026 г. отмечает юбилей Полный кавалер ордена «За заслуги перед Отечеством», академик РАН, выдающийся ученый с мировым именем Иван Иванович Дедов — ведущий клиницист-эндокринолог, педагог, опытный организатор здравоохранения России.

И.И. Дедов родился в Воронежской области, в селе Дмитряшевка Хлевенского района Липецкой области. Окончив в 1958 г. среднюю школу с золотой медалью, он поступает в Воронежский медицинский институт. С 1964 г. И.И. Дедов работает в качестве младшего научного сотрудника лаборатории нейроэндокринологии и группы эндокринологии Института медицинской радиологии АМН СССР в Обнинске. В 1973 г. И.И. Дедова приглашают в Москву, и до 1982 г. он занимает должность старшего научного сотрудника лаборатории экспериментальной эндокринологии Института экспериментальной и клинической онкологии АМН СССР. Докторскую диссертацию защитил в 1976 г. С 1982 по 1988 г. — профессор кафедры факультетской терапии 1‑го лечебного факультета Первого МГМУ им. И.М. Сеченова, а с 1988 г. — заведующий кафедрой эндокринологии того же Института.

Дедовым И.И. создан единственный в России и уникальный в мировой медицине ФГБУ «Эндокринологический национальный центр» (ЭНЦ), объединяющий 7 научно-исследовательских институтов: Институт диабета, Институт клинической эндокринологии, Институт детской эндокринологии, Институт репродуктивной эндокринологии, Институт персонализированной эндокринологии, Институт онкоэндокринологии, Институт образовательной деятельности. С 1989 по 2018 гг. И.И. Дедов являлся директором ЭНЦ — широко известного в России и за ее пределами научно-исследовательского клинико-диагностического лечебного, организационно-методического и педагогического комплекса эндокринологического профиля, выполняя функции Головного учреждения по профилю «Эндокринология». С октября 2018 г. по настоящее время И.И. Дедов является президентом ГНЦ РФ ФГБУ «НМИЦ эндокринологии» Минздрава России. С мая 2025 г. Центру присвоено имя академика Ивана Ивановича Дедова.

Под руководством Дедова И.И. создан реальный «Дом Эндокринологии» для специалистов и пациентов, где нет раздробленности на отдельные дисциплины, как в «других странах»: нейроэндокринология, диабетология, тиреоидология и т.д., — с неизбежными при этом потерями в научной и лечебной деятельности, что позволяет коллективу ЭНЦ успешно участвовать в реализации национального проекта «Здравоохранение» по направлениям «Демография», «Детство», «Онкология»: профилактика и лечение эндокринологических, сердечно-сосудистых, онкологических, включая широкий спектр орфанных и таких социально-значимых заболеваний, как сахарный диабет, йододефицитные заболевания щитовидной железы, ожирение и метаболический синдром. Особый акцент сегодня делается на изучении всех коморбидных состояний и осложнений у лиц с эндокринопатиями (кардиоваскулярные, офтальмологические, хирургические, репродуктивные и др. проблемы).

В Центре ежегодно более 150 тысяч пациентов с самыми сложными заболеваниями получают высококвалифицированную специализированную амбулаторную помощь и более 25 тысяч — стационарное лечение из всех регионов Российской Федерации, стран СНГ и дальнего зарубежья.

Сегодня под руководством И.И. Дедова ЭНЦ реализует в полном объеме возложенные на него государством задачи по организации эндокринологической службы страны, включая развитие в регионах специализированной медицинской помощи разных уровней, научную деятельность, подготовку кадров, организационно-методическое и телемедицинское сопровождение.

В рамках приоритетного национального проекта «Здоровье» Дедовым И.И. была реализована научно обоснованная и экономически просчитанная программа, основанная на «Национальных стандартах» развития специализированной и высокотехнологичной медицинской помощи (ВМП) по эндокринологии в Российской Федерации. Эта программа до настоящего времени позволяет тиражировать высокие медицинские технологии, объединяет первичное звено, муниципальные и региональные (межрегиональные) уровни и федеральный центр в единую систему; создает реальные условия доступности ВМП для населения всех, без исключения, регионов России, играет ключевую роль в народосбережении и консолидации усилий по модернизации здравоохранения нашей страны.

Академик И.И. Дедов уже в течение 40 лет является координатором национальной программы РФ «Борьба с йододефицитными заболеваниями щитовидной железы», в рамках которой в течение 10 лет был успешно реализован межрегиональный контрольно-эпидемиологический проект «Тиромобиль», проводятся работы по мониторингу йододефицитных состояний в регионах РФ и по оценке йодной обеспеченности населения, системный многокомпонентный анализ влияния ключевых зобогенных и антропогенных факторов на состояние здоровья населения страны, разрабатываются персонализированные программы популяционной, групповой и индивидуальной профилактики болезней щитовидной железы в разных регионах нашей страны, а также мониторинг состояния доровья их жителей. Результаты этих долгосрочных исследований легли в основу постановления правительства РФ и национальной программы «О мерах профилактики заболеваний, связанных с дефицитом йода», которые сегодня вновь рассматриваются в Государственной думе РФ.

По инициативе и при непосредственном участии И.И. Дедова с 1996 г. была разработана и реализована федеральная целевая программа (ФЦП) «Сахарный диабет», с 2002 г. входившая в ФЦП «Предупреждение и борьба с социально значимыми заболеваниями». В соответствии с резолюциями ООН и ВОЗ, определивших сахарный диабет как опаснейший вызов мировому сообществу, в рамках программы была модернизирована и создана современная диабетологическая служба России, кардинально изменившая ситуацию в стране и позволившая ей войти в первую десятку стран мира по качеству и доступности специализированной помощи многим миллионам больных сахарным диабетом.

Под руководством Дедова И.И. впервые в России создан и сегодня эффективно функционирует Государственный регистр больных сахарным диабетом, который стал уникальной информационно-аналитической системой диабетологической службы России. Функционируют региональные эндокринологические/диабетологические центры и диспансеры, школы по обучению «обучателей» (врачей и медицинских сестер), а также больных взрослых, детей и их родителей, свыше 200 референс-отделений по лечению диабетической ретино- и нефропатии, диабетической стопы, сердечно-сосудистых осложнений с больных с сахарным диабетом. По инициативе И.И. Дедова впервые в России разработаны мобильные лечебно-диагностические комплексы («Диамобили»), которые позволяют и сегодня проводить скрининговые исследования по оценке реальной распространенности сахарного диабета в регионах РФ, проводить мониторинг состояния здоровья населения в сельских, труднодоступных и отдаленных регионах Российской Федерации.

К фундаментальным работам мирового уровня И.И. Дедова относятся многолетние исследования по генетике, иммуногенетике и гормонально-метаболическим маркерам сахарного диабета. Под его руководством открыты гаплотипы, определяющие как индивидуальные риски, так и риски заболеть сахарным диабетом в этнически разных группах населения Российской Федерации. Эти уникальные данные вошли в Международный реестр иммуногенетических исследований сахарного диабета и позволили организовать в России сеть медико-генетических консультаций для прогноза и мониторинга здоровья в группах риска, что позволило рассчитать финансово-экономические, организационные и социальные составляющие для практического здравоохранения регионов РФ.

Под руководством И.И. Дедова разработаны и внедрены полные инновационные цепочки от геномных проектов до новейших технологий в области диагностики, лечения и профилактики таких социально-значимых болезней эндокринной системы, определяющих медицинскую составляющую демографической ситуации в России, как сахарный диабет, болезни репродуктивной системы и щитовидной железы, опухоли эндокринной системы. Создан целый ряд панелей генов, каждая из которых рассчитана на прицельное, в зависимости от клинической ситуации, секвенирование генов, обеспечивая создание генетического паспорта отдельного человека, семьи, этноса. Врач получил возможность предсказывать и нивелировать риски заболеваний, их раннее выявление и возможность назначать максимально эффективную персонализированную терапию.

В ГНЦ РФ ФГБУ «НМИЦ эндокринологии» под руководством И.И. Дедова уже в течение многих десятилетий реализуется эндокринологическая составляющая национальной программы «Здоровый ребенок», в рамках которой впервые в России проводится неонатальный скрининг трех орфанных заболеваний:

С 2015 г. в новом корпусе ЭНЦ успешно функционируют новые клинико-лабораторные подразделения, отделение вспомогательных репродуктивных технологий, клинические отделения по всему профилю детской эндокринологии, отделение радионуклидной диагностики и терапии, биобанки раритетных биоматериалов, лаборатория КЛЭМП-технологий и персонализированной терапии сахарного диабета, отделения наследственных и орфанных заболеваний, постнатального мониторинга наследственных эндокринопатий, отдел геномных, транскриптомных, протеомных и метаболомных технологий, отдел патоморфологии с лабораторией электронной микроскопии.

В ответ на обращение Дедова И.И. к президенту Российской Федерации Путину В.В., по поручению президента РФ Владимира Путина и на основании постановления правительства РФ №767 от 28 апреля 2022 года ЭНЦ был присвоен статус государственного научного центра (ГНЦ).

Дедовым И.И. впервые в нашей стране была разработана принципиально новая доктрина профилактической (предсказательной) эндокринологии, основанная на геномных, постгеномных, иммунных, гормонально-метаболических и клеточных технологиях. Персонализированная модель эндокринологии позволяет прогнозировать риски болезней и их осложнений, а клинические, молекулярно-генетические и гормонально-метаболические технологии позволяют подобрать лечение персонально каждому больному, а не лечить по шаблону болезнь. Результаты этих исследований, охватывающие ключевые направления современной эндокринологии, диабетологии, онкоэндокринологии, репродуктивной эндокринологии легли в основу рекомендаций, по которым организована работа эндокринологической службы Российской Федерации. За внедрение модели персонализированной медицины в эндокринологии Дедову И.И. была присуждена Государственная премия в области науки и технологий 2017 года.

Впервые в истории благодаря работе уникального отделения вспомогательных репродуктивных технологий, созданного в марте 2009 г. в ЭНЦе по инициативе Дедова И.И., с помощью предимплантационных технологий появилась возможность у людей с наследственными заболеваниями, желающими иметь ребенка, исключить из этапа оплодотворения «больную клетку», несущую ген болезни, и тем самым «прервать» многовековые цепочки наследственных заболеваний и подарить таким семьям счастье рождения здорового ребенка. Таким образом на свет появилось более полутора тысяч здоровых малышей.

Дедов И.И., являясь более 30 лет президентом головного центра по проблеме «Эндокринология», главным специалистом-экспертом эндокринологом и председателем профильной комиссии по эндокринологии Министерства здравоохранения РФ, экспертом ВОЗ по сахарному диабету, президентом Ассоциации эндокринологов России и профессором кафедры эндокринологии Первого Московского государственного медицинского университета им. И.М. Сеченова, вносит большой вклад в развитие медицинской науки, модернизацию эндокринологической и диабетологической службы в России, подготовку высококвалифицированных кадров. По инициативе Дедова И.И. были разработаны и успешно внедрены на кафедрах и курсах эндокринологии ВУЗов РФ уникальные междисциплинарные проблемные программы обучения врачей различных специальностей по разным направлениям клинической эндокринологии. Впервые в нашей стране Дедов И.И. инициировал и рекомендовал для внедрения в программы обучения в клинической ординатуре по профилю «эндокринология» принципы сопровождения, построения и реализации индивидуальных образовательных траекторий обучающихся, т.е. принципы тьюторства в высшей медицинской школе. Под руководством И.И. Дедова впервые в России внедрена специализированная структурированная программа подготовки эндокринологов для регионов РФ по принципу: «от студенческой скамьи до профессорско-преподавательского состава». Была подготовлена целая плеяда докторов медицинских наук, которые стали впоследствии профессорами и руководителями кафедр и курсов эндокринологии в ВУЗах РФ, получили статус главных внештатных специалистов эндокринологов федеральных округов или крупных регионов РФ, что имеет огромное значение для подготовки региональных высокопрофессиональных кадров.

Дедов И.И., являясь Почетным Президентом Общероссийской общественной организации инвалидов «Российская диабетическая ассоциация», объединяющей более 3 миллионов больных и более 37 юридических региональных подразделений, смог консолидировать усилия пациентских организаций, ученых и медицинских работников, организаторов здравоохранения и общества в целом, чтобы объединить эти усилия и иметь возможность ответить на все вызовы, связанные с неинфекционной пандемией сахарного диабета в нашей стране и в мире, достойно решить поставленные перед российским эндокринологическим сообществом задачи и, возможно, найти инновационные персонализированные способы/методы лечения сахарного диабета и предупреждения осложнений на доклинической стадии их развития.

Это послужило началом плодотворной работы в нашей стране по поддержке больных сахарным диабетом и защите их законных прав и интересов. Были направлены официальные обращения к органам законодательной и исполнительной власти России о статусе больного сахарным диабетом, который стал звучать на самом высоком государственном уровне. Вопросы отечественной диабетологии стали чаще рассматриваться на страницах печатных медицинских изданий, на конгрессах и форумах профессиональных ассоциаций и общественных организаций, а также в СМИ. А это дорогого стоит!

Дедовым И.И. опубликовано более 950 научных трудов, из них более 400 — за рубежом, в том числе 85 монографий, учебников, руководств и атласов, клинических рекомендаций для практических врачей.

Дедов И.И. — член Французской академии наук, с 2011 по 2013 гг. — президент Российской академии медицинский наук, с 2014 г. по 2017 г. — вице-президент РАН, с 2017 года и по настоящее время — член президиума РАН, обладатель многих статусных наград, в т.ч. государственных: Заслуженный деятель науки РФ (25 июня 1997 г.), орден «Дружбы народов» (12 сентября 1994 г.), медаль «В память 850-летия Москвы» (26 февраля 1997 г.), полный кавалер ордена «За заслуги перед Отечеством» (19 февраля 2001 г.), (18 октября 2004 г.), (2 февраля 2008 г.), (26 июня 2013 г.), орден «Почета» — февраль 2015 г., лауреат высшей награды Российской академии медицинских наук «Премии и золотой медали им. Н.И. Пирогова» (2011 г.), «Лауреат премии правительства Российской Федерации в области науки и техники» за создание и внедрение в практику здравоохранения Российской Федерации системы современных технологий диагностики, лечения и профилактики сахарного диабета» (27 февраля 2013 г.), орден Франции «Командора за заслуги» (05 декабря 2013 г.).

Лауреат государственной премии Российской Федерации в области науки и технологий за 2017 г. за серию работ по экспериментальной и клинической эндокринологии с научным обоснованием и внедрением в здравоохранение инновационной модели персонализированной (предупредительной) медицины, медаль «За заслуги перед отечественным здравоохранением» (октябрь 2018 г.), орден святителя Луки (архиепископа Крымского) I степени (февраль 2021 г.), Герой Труда Российской Федерации (указ президента РФ №83 от 12.02.2021 г.), благодарность президента РФ (октябрь 2021 г.), лауреат высшей награды Российской академии наук в области медицины «Премии и золотой медали им. Н.И. Пирогова» (2024 г.), Почетная грамота Президента Российской Федерации (13.02.2025 г.).

В сущности, в современной России ключевые инновационные направления фундаментальной и клинической эндокринологии, создание не только ЭНЦа, но и всей эндокринологической службы страны, были инициированы академиком И.И. Дедовым.

В течение всей трудовой деятельности Дедов И.И. уделял огромное внимание изданию научных профессиональных журналов для эндокринологов и врачей смежных специальностей, являясь главным редактором 6 журналов: «Проблемы эндокринологии», «Сахарный диабет», «Вестник репродуктивного здоровья», «Ожирение и метаболизм», «Остеопороз и остеопатии», «Эндокринная хирургия».

Редколлегия и редакционный совет журнала «Проблемы эндокринологии» сердечно поздравляют своего главного редактора и желают ему здоровья, полного благополучия и долгих плодотворных творческих лет на благо отечественной эндокринологии.

